# Assessing the Validity of Diffusion Weighted Imaging Models: A Study in Patients with Post-Surgical Lower-Grade Glioma

**DOI:** 10.3390/jcm14020551

**Published:** 2025-01-16

**Authors:** Anouk van der Hoorn, Lesley E. Manusiwa, Hiske L. van der Weide, Peter F. Sinnige, Rients B. Huitema, Charlotte L. Brouwer, Justyna Klos, Ronald J. H. Borra, Rudi A. J. O. Dierckx, Sandra E. Rakers, Anne M. Buunk, Joke M. Spikman, Remco J. Renken, Ingeborg Bosma, Roelien H. Enting, Miranda C. A. Kramer, Chris W. J. van der Weijden

**Affiliations:** 1 Department of Radiology, Medical Imaging Center, University Medical Center Groningen, University of Groningen, 9713 GZ Groningen, The Netherlands; 2 Department of Radiation Oncology, University Medical Center Groningen, University of Groningen, 9713 GZ Groningen, The Netherlands; 3 Department of Neurology, University Medical Center Groningen, University of Groningen, 9713 GZ Groningen, The Netherlands; 4 Department of Nuclear Medicine and Molecular Imaging, Medical Imaging Center, 9713 GZ Groningen, The Netherlands; 5 Department of Biomedical Sciences of Cells & Systems, Cognitive Neuroscience Center, University Medical Center Groningen, University of Groningen, 9713 GZ Groningen, The Netherlands

**Keywords:** diffusion tensor imaging, diffusion kurtosis imaging, white matter tract integrity, neurite orientation dispersion and density imaging, fixel-based analysis

## Abstract

**Background:** Diffusion weighted imaging (DWI) is used for monitoring purposes for lower-grade glioma (LGG). While the apparent diffusion coefficient (ADC) is clinically used, various DWI models have been developed to better understand the micro-environment. However, the validity of these models and how they relate to each other is currently unknown. Therefore, this study assesses the validity and agreement of these models. **Methods:** Fourteen post-treatment LGG patients and six healthy controls (HC) underwent DWI MRI on a 3T MRI scanner. DWI processing included diffusion tensor imaging (DTI), diffusion kurtosis imaging (DKI), white matter tract integrity (WMTI), neurite orientation dispersion and density imaging (NODDI), and fixel-based analysis (FBA). Validity was assessed by delineating surgical cavity, peri-surgical cavity, and normal-appearing white matter (NAWM) in LGG patients, and white matter (WM) in HC. Spearman correlation assessed the agreement between DWI parameters. **Results:** All obtained parameters differed significantly across tissue types. Remarkably, WMTI showed that intra-axonal diffusivity was high in the surgical cavity and low in NAWM and WM. Most DWI parameters correlated well with each other, except for WMTI-derived intra-axonal diffusivity. **Conclusion:** This study shows that all parameters relevant for tumour monitoring and DWI-derived parameters for axonal fibre-bundle integrity (except WMTI-IAS-D_a_) could be used interchangeably, enhancing inter-DWI model interpretability.

## 1. Introduction

Lower-grade glioma (LGG) is a primary brain tumour with relatively high patient survival rates [1]. LGG occurs in one to two cases per 100,000 people yearly [2,3]. Negative prognostic factors include large tumour volume, incomplete surgical resection, IDH wild-type status, p53, an absence of 1p/19q codeletion, and a lack of MGMT promoter methylation [4]. Due to treatment improvements, life expectancy increases, making it more important to consider the treatment effects on patients’ quality of life. This is especially relevant since research suggests that radiotherapy causes long-term cognitive deficits [5]. Diffusion weighted imaging (DWI) with magnetic resonance imaging (MRI) is often performed prior, during, and after treatment to monitor the tumour response, tumour progression, and treatment-related side effects in otherwise healthy brain tissue [6]. Various models have been developed for DWI processing to extract more information from the DWI scans, all with significant overlap but also specific differences. For a proper understanding of treatment-induced DWI changes in patients with brain tumour, it is essential to understand to what extent these models give different answers.

DWI measures Brownian motion, which is the random intrinsic motion of molecules with a net displacement of zero [7]. This random motion is limited upon an increase in cellular density due to physical constraints, which results in a reduced Brownian motion in tumours. In contrast, in non-tumorous tissue, the direction of the Brownian motion within the brain is primarily governed by axons, resulting in more Brownian motion parallel to the axons, and reduced Brownian motion perpendicular to the axons [7]. Several DWI models have been developed to quantify this Brownian motion. For this, it is important to differentiate between hindered diffusion and restricted diffusion. Hindered diffusion is restricted Brownian motion due to cellular structures, which can be measured with low b-values (b < 2000 s/mm^2^) [8]. However, when higher b-values are used (b > 2000 s/mm^2^), one also measures restricted diffusion, which is the Brownian motion of water molecules that are highly restricted in their movement, to a much larger extent than hindered diffusion, due to confinement within specific compartments or structures (e.g., organelles or cellular components).

From the various DWI models, diffusion tensor imaging (DTI) and diffusion kurtosis imaging (DKI) describe the Brownian motion in relation to the directions, e.g., axial diffusivity (AD), radial diffusivity (RD), mean diffusivity (MD), and fractional anisotropy (FA) [9,10]. DKI requires the acquisition of a high b-value (b ≥ 2000 s/mm^2^), which also enables one to estimate the effect of restricted diffusion on the diffusion direction, called kurtosis [10]. With DKI, additional parameters like axial kurtosis (AK), radial kurtosis (RK), mean kurtosis (MK), kurtosis fractional anisotropy (KFA), and mean kurtosis tensor (MKT) are generated. DTI and DKI are both affected by crossing fibres, leading to altered interpretations of the tissue micro-structure, more advanced methods that account for crossing fibres have been developed.

With DWI models other than DTI and DKI, one can perform white matter tract integrity (WMTI), neurite orientation dispersion and density imaging (NODDI), and fixel-based analysis (FBA) [11,12,13]. These DWI models require the acquisition of at least one high b-value (in combination with two low b-values, e.g., b = 0 s/mm^2^ and b = 1000 s/mm^2^) [11,12,13]. As the name indicates, WMTI is specifically developed for the assessment of white matter and, as such, does not account in the modelling for cerebral spinal fluid (CSF). The parameters generated with WMTI are intra-axonal space diffusivity (WMTI-IAS-D_a_), extra-axonal space axial diffusivity (WMTI-EAS-AD), extra-axonal space radial diffusivity (WMTI-EAS-RD), extra-axonal space mean diffusivity (WMTI-EAS-MD), axonal water fraction (WMTI-AWF), and extra-cellular tortuosity (WMTI-EAS-Tort), which quantifies the complexity of the extra-cellular microenvironment, reflecting how much water movement is restricted by barriers within the tissue [11]. In contrast to WMTI, NODDI actually accounts for CSF, leading to the generation of the parameters intra-cellular volume fraction (NODDI-FICVF), isotropic volume faction (NODDI-FISO), and the orientation dispersion index (NODDI-ODI), which describes the degree of dispersions of axons within a voxel [12]. With FBA, the fibre bundles in a voxel, called fixel, can be characterized according to the fibre density (FBA-FD), fibre-bundle cross-section (FBA-FC), and the combination of the two-fibre-bundle density and cross-section (FBA-FDC) [13].

DWI MRI is used in the routine follow-up of patients with brain tumours, including LGG. There are many different methods with which to analyze the DWI data, of which ADC is the most commonly used within our clinic. However, more micro-structural specific parameters can be generated from DWI data using either DTI, DKI, FBA, WMTI, or NODDI. The validity and correspondence of these different models have never been thoroughly assessed. Therefore, this study aimed to determine the validity of these methods in patients with post-treatment LGG and assess the agreement between the methods.

## 2. Methods

### 2.1. Theoretical Background of DWI Models

Grossly, DWI models (Table 1) can be separated into two classes: those focusing on hindered diffusion only, and those that account for both hindered and restricted diffusion. Generally, the ones focused on hindered diffusion only are using b-values equal to or less than 1000 s/mm^2^; the diffusion is then considered to be unaffected by restrictive components.

The apparent diffusion coefficient (ADC) is currently the most routinely used DWI parameter within our clinic. The ADC reflects the change in signal intensity of DWI due to the applied b-value often compared to b = 0 s/mm^2^, and is as such a parameter for the amount of diffusion. When DWI is acquired with b = 0 s/mm^2^ as reference and b = 1000 s/mm^2^ for diffusion sensitivity, thus without either very low- or high b-values (0 > b < 200 s/mm^2^ and b > 2000 s/mm^2^, respectively), the ADC is considered to represent pure diffusion. The ADC can be obtained per voxel via the following formula:

$$ADC=-\frac{\ln\left(\frac{S_{2}}{S_{1}}\right)}{\left(b_{2}-b_{1}\right)}$$

In this formula, S_1_ is the signal intensity of the image with lowest b-value acquired (e.g., b = 0 s/mm^2^), S_2_ is the signal intensity of the image with highest b-value acquired (e.g., b = 1000 s/mm^2^), b_1_ is the b-value corresponding to the image of S_1_, and b_2_ is the b-value corresponding to the image of S_2_ [14,15]. On the ADC images, areas with high diffusion appear dark (indicating fast diffusion), while areas of restricted diffusion appear bright (indicating slow diffusion).

With DTI, the directions of Brownian motion can be estimated. As such, the axial- (DTI-AD), radial- (DTI-RD), mean-diffusivity (DTI-MD), and fractional anisotropy (DTI-FA) can be generated [16,17]. However, because DTI is generated with low b-values, it only takes hindered diffusion into account. As this is less biologically representative, because diffusion can both be hindered and restricted, DKI accounts for both and therefore necessitates the acquirement of high b-values (b ≥ 2000 s/mm^2^) [10]. Therefore, DKI generates parameters similar to DTI, but then correct them for the kurtosis effects caused by the restricted diffusion.

While both DTI and DKI already provide more insight into the micro-structure than ADC, the estimates of DTI and DKI are affected by the presence of crossing fibres. For example, a low FA value can be obtained when fibres intersect each other, but also when there are few to no fibres. More advanced DWI models like WMTI, NODDI, and FBA can account for the effect of crossing fibres.

According to WMTI, the brain tissue can be separated into two different compartments, the intra-axonal space (IAS) and the extra-axonal space (EAS) [11]. The diffusivity can be measured from both IAS and EAS, but the contribution of transversal diffusivity in IAS is negligible due to the small diameter of axons. Therefore, IAS is dominated by axial diffusivity, and the trace of the eigenvalues is used to determine the axonal diffusion coefficient (D_a_). For EAS, the transversal diffusivity is non-negligible, and hence the axial (WMTI-EAS-AD), radial (WMTI-EAS-RD), and mean diffusivity (WMTI-EAS-MD) can be estimated. The axonal water fraction (WMTI-AWF) can be estimated from the diffusivity and kurtosis parameters, as described by Fieremans et al. in Equation (8) [11].

With NODDI, the DWI signal can be separated into 3 compartments: intra-neurite (neurite is the combination of a dendrite and axon), extra-neurite, and free water [12]. This would lead to the generation of the neurite density index (NDI), also known as the intra-cellular volume fraction, (NODDI-FICVF) and free water fraction (FWF), also known as the isotropic volume fraction (NODDI-FISO). NODDI-FICVF is intra-neurite diffusion divided by the summation of intra- and extra-neurite diffusion. NODDI-FISO is the free water diffusion divided by the summation of free water, intra-neurite, and extra-neurite diffusion.

FBA considers that a voxel can consist of multiple fibre populations with different directions [13]. This can subsequently be quantified by calculating the fibre orientation distribution per voxel. The FOD captures the presence and orientation of multiple fibre populations (including crossing fibres) within each voxel. From the FOD images, parameters like fibre density (FBA-FD), fibre-bundle cross-section (FBA-FC), and fibre density and cross-section (FBA-FDC) can be generated. FBA-FD reflects the axonal packing density within a fibre population, whereas FBA-FC quantifies the changes in cross-section of a fibre bundle as it traverses through the brain.

### 2.2. Subjects

Seventeen patients with post-treatment LGG and six healthy controls (HCs) were included in this cross-sectional prospective study. Treatment consisted of surgery, photon radiotherapy, and chemotherapy. Inclusion criteria for HC were age ≥18 years and absence of neurological or psychiatric disorders. Inclusion criteria for patients with post-treatment LGG were tumour diagnosis during adulthood (≥18 years), LGG diagnosis, at least one year after completion of radiotherapy, and without signs of tumour progression. LGG diagnosis was described as either isocitrate dehydrogenase (IDH) mutated astrocytoma, World Health Organization (WHO) grade 2, or oligodendroglioma WHO grade 2 and 3. The following exclusion criteria were applied for both HC and LGG patients: pregnancy or breastfeeding; a previous adverse reaction to gadolinium; claustrophobia; a diagnosis of cerebrovascular disease; and the presence of magnetizable materials in the body. Written informed consent was obtained from all study participants. This study was approved by the Medical Ethics Review Committee of the University Medical Center Groningen (UMCG) in accordance with the Helsinki Declaration.

### 2.3. Data Acquisition

All scans were acquired using the same 3.0 Tesla Siemens Magnetom Prisma MRI scanner (Siemens Healthineers, Erlangen, Germany) equipped with a 64-channel head coil. The brain imaging protocol included the following sequences: a sagittal 3D T_1_w MP-RAGE (TR: 2300 ms; TE: 2.31 ms; TI: 900 ms; flip angle: 8°; voxel size: 0.9 × 0.9 × 0.9 mm, TA: 6:35), a sagittal 3D T_2_w-FLAIR SPACE (TR: 5000 ms; TE: 392 ms; TI: 1800 ms; flip angle: 90°, voxel size: 0.9 × 0.9 × 0.9 mm, TA: 6:22), a DWI EPI (TR: 2200 ms; TE: 77 ms; flip angle: 90°; 60 transversal slices; AP phase encoding direction; 64 diffusion directions, b-values 0, 1000, and 2500 s/mm^2^; voxel size: 2.5 × 2.5 × 2.5 mm, partial Fourier 6/8, SMS acceleration factor slice 4, TA: 5:07), and a DWI (TR: 2200 ms; TE: 77 ms; flip angle: 90°; 60 transversal slices; PA phase encoding direction; 64 diffusion directions, b-values 0 s/mm^2^; slice thickness: 2.5 mm; and voxel size: 2.5 × 2.5 × 2.5 mm, partial Fourier 6/8, SMS acceleration factor slice 4, TA: 0:51).

### 2.4. Image Data Processing

Prior to modelling the DWI, the DWI data were first corrected for noise and Gibbs ringing artefacts, followed by a field map correction of susceptibility distortions, motion correction with correction for eddy–current-induced distortions, and bias field correction [18,19,20,21,22,23,24,25,26]. These corrected diffusion data were then used for the modelling of DTI using b = 0 and b = 1000 s/mm^2^; modelling for DKI, WMTI, NODDI, and FBA were performed using b = 0, b = 1000, and b = 2500 s/mm^2^. DTI was performed using the FMRIB software library (FSL v6.0.6.5) [22]. DKI with diffusion kurtosis estimator (DKE v2.6.0) [27]. WMTI with Python diffusion parameter estimation with Gibbs and noise removal (PyDESIGNER v1.0) [28]. NODDI with the NODDI toolbox (v1.05) [12]. and FBA with MRtrix v3.0 [29]. For NODDI, two models were applied, with the differences that axons were either represented as sticks or as cylinders. While sticks is the most common method, cylinders might be more biologically representative as axons have a diameter and intra-cellular fluid [30]. Subsequently, all the processed DWI data were co-registered using FSL to obtain Gibbs ringing artefact and bias field-corrected T_1_w images.

### 2.5. ROI Definition

On T_2_w-FLAIR, the surgical cavity, the peri-surgical cavity, as determined by hyper-intense area on the T_2_w-FLAIR around the surgical cavity, and a 0.5 mL sphere of normal-appearing white matter (NAWM) crosswise to the surgical cavity were delineated on the MRI scans of the patients with LGG. The white matter (WM) of HCs was obtained after the tissue segmentation of T_1_w with SPM12, using a probability threshold of 0.5. Brain parcellation with the Hammers atlas was performed for all subjects undergoing T_1_w MRI. For subsequent statistical analysis, only Hammers atlas ROIs of the unaffected hemisphere of patients with LGG were considered.

### 2.6. Validity Assessment

Within the surgical cavity, more than 1 year after surgery, we would expect an absence of axons due to the surgical removal of tissue, and therefore axonal fibre-bundle integrity parameters should have no values within the surgical cavity. In contrast, within intact white matter tissue, a high number of axons is expected. Therefore, the axonal fibre-bundle integrity parameters should have high values within the white matter. For axial, radial, and mean diffusivity, one would expect a different behaviour within the surgical cavity and white matter. Due to the absence of tissue, the water molecules are not restricted in their motion, and hence high AD, RD, and MD values are expected in the surgical cavity, whereas low AD, RD, and MD values are expected in the white matter, due to the high cellular density. The peri-surgical cavity is expected to be in the middle, as the edema observed with T_2_w-FLAIR decreases the cellular density.

### 2.7. Statistical Analysis

Normality was assessed using the Kolmogorov–Smirnov test, for which a *p* < 0.05 indicated a non-normal (or non-parametric) distribution of the data. Subsequently, comparisons were performed with either ANOVA or Kruskal–Wallis tests depending on normality, and corrected for multiple comparisons using Bonferroni corrections. For correlation analysis, Spearman was used as Spearman is less sensitive to outliers than Pearson. Subsequently, the ICC was calculated using the mixed model with absolute agreement. All of the statistical analyses were performed with SPSS Statistics, version 23 (IBM, Chicago IL, USA). A two-sided *p*-value of <0.05 was used for all comparisons.

## 3. Results

### 3.1. Demographics

We included 14 patients with post-surgery LGG and 6 HCs in this study. Three of the initial seventeen patients with LGG were excluded to avoid inter-scanner variability as the MRIs were not acquired on the 3T Prisma Siemens scanner. The demographics of the subjects are provided in Table 2. Age and gender did not significantly differ between patients and HCs (U = 31, *p* = 0.397; T = 0.379, *p* = 0.709, respectively).

### 3.2. Assessment of Axonal Fibre-Bundle Integrity Parameters

**Figure 1:**
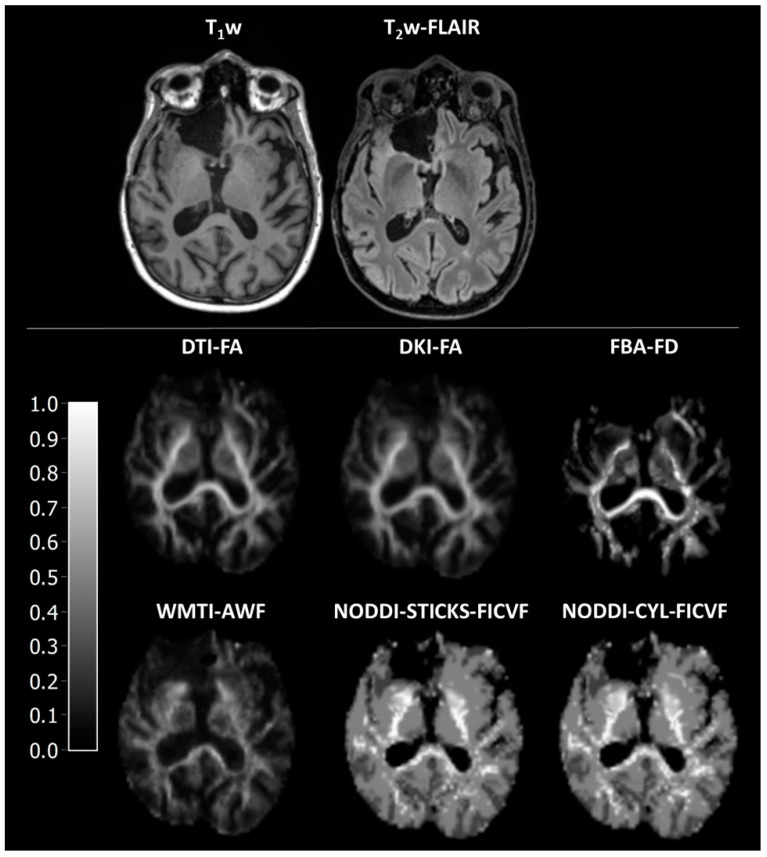
Representative images of axonal fibre-bundle integrity parameters. DTI-FA is fractional anisotropy as estimated using diffusion tensor imaging, DKI-FA is fractional anisotropy as estimated using diffusion kurtosis imaging, FBA-FD is fibre density as estimated using fixel-based analysis, WMIT-AWF is axonal water fraction as estimated with white matter tract integrity, NODDI-STICKS-FICVF is the intracellular volume fraction as estimated using the neurite orientation dispersion and density imaging with the sticks model for neurites, and NODDI-CYL-FICVF is the intracellular volume fraction as estimated using the neurite orientation dispersion and density imaging with the cylinder model for neurites.

**Figure 2:**
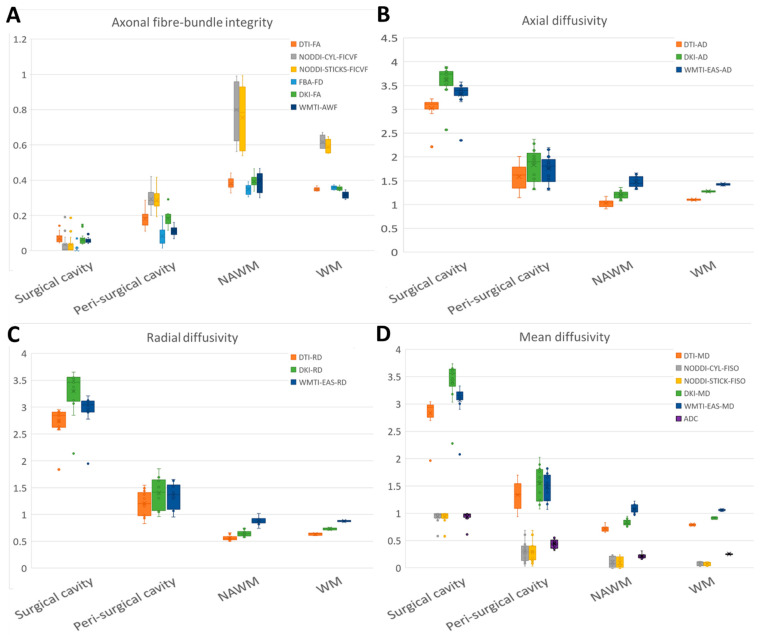
DWI-derived parameters across different tissue types. WMTI-IAS-D_a_ is excluded from the images due to the extreme different values for axonal fibre-bundle integrity and can be found in Supplementary Figure S1. Abbreviations: NAWM = normal-appearing white matter, WM = white matter, DTI = diffusion tensor imaging, DKI = diffusion kurtosis imaging, WMTI = white matter tract integrity, NODDI-CYL = neurite orientation dispersion and density imaging with cylindric model, NODDI-STICKS = neurite orientation dispersion and density imaging with sticks model, FBA = fixel-based analysis, AD = axial diffusivity, EAS = extra-axonal space, IAS = intra-axonal space, AWF = axonal water fraction, FD = fibre density, FICVF = intra-cellular volume fraction, RD = radial diffusivity, FISO= isotropic volume fraction, MD = mean diffusivity, ADC = apparent diffusion coefficient, FA = fractional anisotropy.

As a sanity check to assess whether the various methods displayed discrepant results in correspondence with the different tissue composure, the values of the various parameters were compared between surgical cavity, peri-surgical cavity, NAWM, and WM. As expected, all parameters were significantly different between different tissues (H = 40.4–42.2, *p* < 0.001, Figure 1 and Figure 2A, Supplementary Tables S1 and S2, Supplementary Figures S1 and S2). Aside from WMTI-IAS-D_a_, all parameters had a gradient of low/absent in the surgical cavity, higher in peri-surgical cavity, and highest in NAWM and WM (Supplementary Tables S2 and S3).

**Figure 3:**
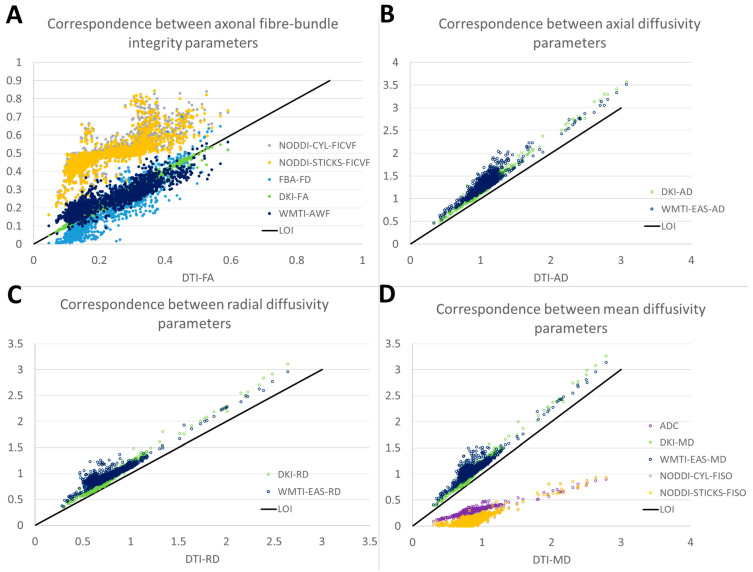
Correspondence assessment between the various DWI-derived parameters. WMTI-IAS-D_a_ is excluded from the images due to the extremely different values for axonal fibre-bundle integrity, and can be found in Supplementary Figure S3. For abbreviations, see Figure 2.

To determine the agreement between the various DWI methods, the correspondence between them was assessed. All methods strongly correlated with each other (r = 0.77–1.00, Figure 3A, Supplementary Table S4), except for WMTI intra-axonal diffusivity (r = 0.05–0.30). Furthermore, a low bias as depicted by differences in slope and ICC was observed (Figure 3A and Supplementary Table S5) between DTI-FA, DKI-FA, WMTI-AWF, and FBA-FD, and between NODDI-STICKS-FICVF and NODDI-CYL-FICVF, but with a high bias between the two groups. When compared to each other, the methods were significantly different from each other at a group level (H = 9609, *p* < 0.001, Table 3, Supplementary Tables S2 and S6). Bonferroni-corrected post hoc tests showed that all were different except for DTI-FA from DKI-FA (H = 126, *p* = 1.000), NODDI-CYL-FICVF from NODDI-STICK-FICVF (H = 162, *p* = 1.000), and DKI-FA from WMTI-AWF (H = −357, *p* = 0.076), as determined via Bonferroni-corrected post hoc tests. These findings suggest that while the absolute values were statistically different from each other, the relative change in parameters between the different ROIs was similar for all axonal fibre-bundle integrity parameters (except WMTI-IAS-D_a_).

### 3.3. Assessment of Axial Diffusivity-Related Parameters

As expected, also for the axial diffusivity, all parameters were significantly different between different tissues (H = 32.7–42.3, *p* < 0.001, Figure 2B and Supplementary Figure S4, Supplementary Table S1), with a high gradient in the surgical cavity, a lower gradient in the peri-surgical cavity, and the lowest gradient in NAWM and WM (Supplementary Tables S2 and S7).

All methods strongly correlated with each other (r = 0.81–0.97, Figure 3B, Supplementary Table S8), with a low bias between each other, as depicted by the slope and ICC (Figure 3B, Supplementary Table S9). The values of DKI-AD and WMTI-EAS-AD seem to be highly similar, but slightly higher than the values of DTI-AD (Figure 3B). When compared to each other, all methods were significantly different from each other (H = 2059, *p* > 0.001, Table 3, Supplementary Tables S2 and S6), further supported by Bonferroni-corrected post hoc tests (Supplementary Table S6). Both DKI-AD and WMTI-EAS-AD have higher values than DTI-AD, whereas DKI-AD seems to have higher estimates than WMTI-EAS-AD for high AD estimates and lower estimates than WMTI-EAS-AD for low AD estimates. These findings suggest that while the absolute values were statistically different from each other, the relative change in parameters between the different ROIs was similar for all AD-related parameters.

### 3.4. Assessment of Radial Diffusivity Related Parameters

As expected, the radial diffusivity parameters were all significantly different between different tissues (H = 41.1–42.9, *p* < 0.001, Figure 2C and Supplementary Figure S5, Supplementary Table S1), with a high gradient in the surgical cavity, a lower one in peri-surgical cavity, and the lowest one in NAWM and WM (Supplementary Tables S2 and S10).

All methods strongly correlated with each other (r = 0.85–0.98, Figure 3C, Supplementary Tables S2 and S11), with a low bias between each other, as depicted by the slope and ICC (Figure 3C, Supplementary Table S12). The values of DKI-RD and WMTI-EAS-RD seem to be a bit higher than DTI-RD (Figure 3C). When compared to each other, all methods were significantly different from each other (H = 1406, *p* < 0.001, Table 3, Supplementary Table S6), further supported by Bonferroni-corrected post hoc tests (Supplementary Table S6). Both DKI-RD and WMTI-EAS-RD have higher values than DTI-RD. For high RD values, DKI-RD seems to have higher estimates than WMTI-EAS-RD, whereas for low RD values, DKI-RD has lower estimates than WMTI-EAS-RD. These findings suggest that while the absolute values were statistically different from each other, the relative change in parameters between the different ROIs was similar for RD-related parameters.

### 3.5. Assessment of Mean Diffusivity-Related Parameters

**Figure 4:**
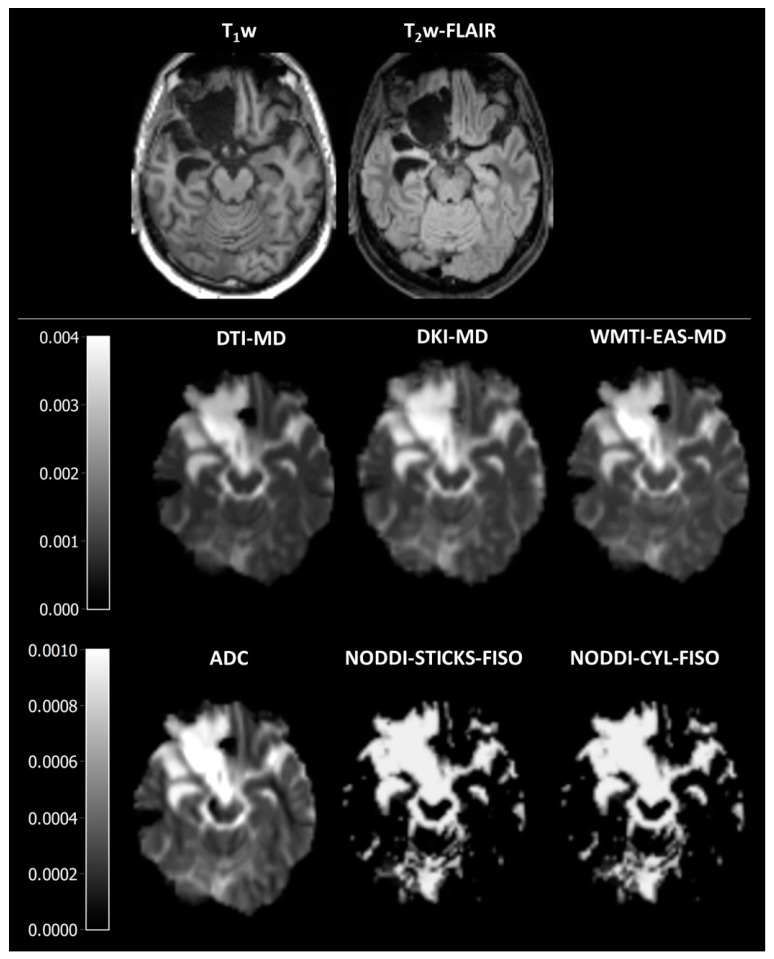
Representative images of mean diffusivity-related parameters. DTI-MD is the mean diffusivity as estimated using diffusion tensor imaging, DKI-MD is the mean diffusivity as estimated using diffusion kurtosis imaging, WMTI-EAS-MD is the extra-axonal space mean diffusivity as estimated using white matter tract integrity, ADC is the apparent diffusion coefficient, NODDI-STICKS-FISO is the isotropic volume fraction as estimated using neurite orientation dispersion and density imaging with the sticks model for neurites, and NODDI-CYL-FISO is the isotropic volume fraction as estimated using neurite orientation dispersion and density imaging with the cylinder model for neurites.

Similarly, as expected, all parameters of the mean diffusivity were significantly different between different tissues (H = 34.9–42.9, *p* > 0.001, Figure 2D and Figure 4, Supplementary Table S1), with a high gradient in the surgical cavity, a lower one in the peri-surgical cavity, and the lowest one in NAWM and WM (Supplementary Tables S2 and S13).

All methods strongly correlated with each other (r = 0.61–0.99, Figure 3D, Supplementary Table S14). Furthermore, a low bias was observed, as depicted by differences in slope and ICC (Figure 3D and Supplementary Table S15) between DTI-MD, DKI-MD, and WMTI-EAS-MD, and between ADC, NODDI-STICKS-FISO, and NODDI-CYL-FISO, but a high bias between the two groups was noticed. When compared to each other, all methods were significantly different to each other (H = 9506, *p* > 0.001, Table 3, Supplementary Table S6), except for NODDI-CYL-FISO from NODDI-STICKS-FISO (H = −14, *p* = 1.000). Both DKI-MD and WMTI-EAS-MD have higher values than DTI-MD, whereas DKI-MD seems to have higher estimates than WMTI-EAS-MD for high MD estimates and lower estimates than WMTI-EAS-MD for low MD estimates. Furthermore, both NODDI-CYL-FISO and NODDI-STICKS-FISO have lower MD values than either DTI-MD, DKI-MD, or WMTI-EAS-MD. These findings suggest that while the absolute values were statistically different from each other, the relative change in parameters between the different ROIs was similar for all MD-related parameters.

## 4. Discussion

This study is the first study to assess six different DWI models, ADC, DTI, DKI, WMTI, NODDI, and FBA, within one dataset. We showed that all methods displaying axial-, radial-, or mean-diffusivity, or axonal fibre-bundle integrity, had a high correspondence with each other, except for WMTI-IAS-D_a_. Low bias was observed in the axial and radial diffusivity-related parameters, and higher bias was observed between the axonal fibre-bundle integrity and mean diffusivity-related parameters. All measurements were also significantly different from each other, except for axonal fibre-bundle integrity DTI-FA from DKI-FA, DTI-FA from WMTI-AWF, and NODDI-CYL-FICVF from NODDI-STICKS-FICVF, and for mean diffusivity NODDI-CYL-FISO from NODDI-STICKS-FISO. Furthermore, all measurements also displayed the expected physiological differences for axial-, radial-, and mean-diffusivity, and axonal fibre-bundle integrity, except for WMTI-IAS-D_a_, which unexpectedly achieves a high intra-axonal axial diffusivity in the surgical cavity.

In general, the different DWI models have a high correspondence with each other, as displayed by the strong correlations. The only discrepant result is in this case WMTI-IAS-D_a_, which is the intra-axonal diffusivity. This parameter is high in the surgical cavity, medium-high in the peri-surgical cavity, and low in the NAWM and WM. As this parameter is supposed to depict the diffusivity within axons, and no axons are present in the surgical cavity, and there is a high abundance in the WM, this finding is rather unexpected. In the documentation of PyDESIGNER, there is no information regarding the calculations of WMTI [28], and neither is there in the original DESIGNER article [31]. However, in the DESIGNER article, the authors cite Fieremans et al. for the WMTI model [11,31]. Therefore, we assume that the developers of PyDESIGNER used the Fieremans model. In the study of Fieremans et al., WMTI-IAS-D_a_ is the trace of the intra-axonal diffusivity, thus the summation of the intra-axonal eigenvalues [11]. While the article of Fieremans shows similar patterns of WMTI-AWF and WMTI-IAS-D_a_, this correspondence is clearly absent in the results of PyDESIGNER. This difference suggests an erroneous implementation of the WMTI-IAS-D_a_ calculation in PyDESIGNER (Supplementary Figures S1 and S2). Surprisingly, WMTI-AWF generated with PyDESIGNER corresponds with physiological expectations and also with axonal fibre-bundle integrity parameters derived from other DWI models (Figure 1 and Figure 2).

Interestingly, the DKI-derived parameters correspond well with both DTI and WMTI (except for WMTI-IAS-D_a_), whereas DTI corresponds only moderately with WMTI. This suggests that kurtosis has a high contribution to the moderate correspondence between DTI and WMTI parameters, as the DKI-derived parameters, which are corrected for kurtosis, have a high correspondence with both. The high correspondence between DTI-FA and DKI-FA suggests that the kurtosis-corrected diffusivity parameters have the same relation to each other. This is an interesting finding, as the different diffusion directions have the same magnitude of kurtosis. As kurtosis is governed by restricted diffusion due to confinement within specific compartments or structures, this confinement seems to be consistent across diffusivity directions. Therefore, the equal diffusion restriction in each direction suggests a homogenous tissue composure within voxels in each direction. Furthermore, in contrast to the often-posed statement that DTI-MD and ADC are the same, our study shows highly discrepant values. Nonetheless, there is a strong correlation between the two parameters. However, there is significant bias, that is, the values of DTI-MD are consistently higher than the values derived from ADC. Surprisingly, the bias between ADC and either NODDI-STICKS-FISO or NODDI-CYL-FISO was considerably lower. This implies that for certain types of tissue micro-structure or pathology, the NODDI technique, with its ability to distinguish between isotropic and anisotropic diffusion, might offer more relevant insights into the underlying tissue properties influencing ADC compared to the information provided by DTI. It is noteworthy that (either high or low) bias is consistent across subjects. When the bias is consistent between subjects, one can use the various parameters with a simple calibration factor to go from one to the other. This means that for clinical applications, there are no major differences in diagnostic outcomes, and that the methods can be used interchangeably. Another aspect that was remarkable was the high correspondence of NODDI between either the use of cylinders or sticks as a model for axons. We anticipated cylinders to be more biologically representative of axons, due to the diameter, which sticks lack. However, for any measurement, we found high agreement between the usage of either cylinders or sticks. Despite the usage of the cylinder model being, from a theoretical perspective, potentially more biologically representative, there is almost no discrepancy in NODDI parameter estimation due to the underlaying selected tissue model. NODDI-derived parameters, particularly the fraction of intra-cellular volume (FICVF), provide deeper insights into tissue micro-structure by separating isotropic and anisotropic diffusion, allowing for more a specific characterization of tissue properties compared to models like DKI or WMTI. This ability to disentangle free water and neurite density explains why NODDI-FISIO is highly accurate in the surgical cavities, where isotropic diffusion (e.g., from edema or free water) dominates. By directly quantifying neurite density, FICVF offers physiological parameters that reflect changes in tissue integrity, making NODDI particularly suited for understanding pathological processes like edema, gliosis, or tissue remodelling, which traditional models may not capture with the same specificity.

Previous works also showed a high agreement between DTI-FA and DKI-FA [8]. This study performed an analysis in patients with multiple sclerosis (MS), which suggests that the findings are robust across pathologies, as we assessed both patients with post-surgery LGG and HCs. The agreement between DWI-derived parameters should be assessed across multiple pathologies to determine whether the parameter estimations are indeed robust. Commonly, studies assess the diffusivity of regions typified by pathology (e.g., brain tumours, MS lesions, etc.). In our study, we specifically assessed the diffusivity patterns in the surgical cavity, the peri-surgical cavity, NAWM, and WM. To our knowledge, this is the first study assessing the diffusivity in the surgical cavity and the peri-surgical cavity. Therefore, we can only compare our NAWM and WM results with the results of other studies. The DKI and NODDI parameters in NAWM of brain tumour patients by Maximov et al. are comparable with the ones obtained in our study [32]. The DTI, DKI, and FBA parameters in NAWM are also comparable to the ones obtained by van der Weijden et al. [8]. In addition, the study of Piper et al. also showed comparable results for DTI parameters in the NAWM of patients with LGG [33]. While the peri-surgical cavity is not the same as the peri-tumour WM, due to the absence of a tumour, some studies assessed the peri-tumour and found it to be comparable, for DTI [34] and DKI [35] results, with the ones we obtained in the peri-surgical cavity, but with deviant results in ADC [34]. These deviant results for ADC might be due to differences in pulse sequence timing, spatial orientation, bulk tissue motion, etc., which affects ADC and hence makes ADC a less suitable option for tissue quantification.

To our knowledge, this is the first article comparing head-to-head the six different DWI models in a single dataset. A previous study in MS patients assessed the agreement between DTI, DKI, and FBA, and was the first study assessing these three DWI models in a single dataset [8]. The focus of that study was primarily on axonal fibre-bundle integrity parameters, and the authors found high agreement between DTI-FA, DKI-FA, and FBA-FD, which corresponds to the findings of this study.

The major limitation of this study is the usage of only one high b-value (b = 2500 s/mm^2^), as increasing the number of high b-values can enhance the accuracy and robustness of the DKI, FBA, WMTI, and NODDI parameter estimations. Incorporating additional high b-values (e.g., 1500, 2000, 3000 s/mm^2^) would provide more comprehensive sampling of the diffusion signal, improving the separation of linear diffusion and non-Gaussian effects for DKI and refining the compartmentalized signal decomposition critical for NODDI. However, this improvement comes with a significant trade-off: increased image acquisition time. The short total acquisition time of 6 min used in this study reflects a clinically acceptable balance between scan efficiency and model accuracy, ensuring the relevance of parameter generation for clinical settings where patient tolerance and time constraints are critical. Importantly, this study demonstrates that the robust estimation of parameters is achievable with this streamlined protocol, particularly for DKI and WMTI, though the NODDI-derived parameters show greater deviation compared to the other models. This highlights the potential for further optimization in NODDI applications, while affirming the practicality of the current approach for broader clinical utility. Another limitation is the lack of histopathological data to assess the micro-structural content for defining the signal sources. This is especially relevant for the axonal fibre-bundle integrity parameters. However, studies showed that the sources of DWI signals vary across pathologies, hence correspondence between DWI parameters with histology found in one pathology cannot be easily translated to another pathology [36]. Another limitation is the lack of histopathological correlation using biopsies or post-mortem MRI. Histopathological assessment of biopsies requires opening the skull and subsequently the removal of brain tissue. As this is a rather invasive procedure, this is rarely performed in routine clinical care. Nonetheless, a previous study showed strong correlations between diffusion and histopathology from biopsies [37]. The usage of post-mortem MRI to be able to compare imaging findings with histopathology would be severely confounded by tissue alterations that occur due to an absence of an active blood circulation. Therefore, the findings of comparing post-mortem MRI with histopathology can hardly be translated to living patients [38]. Due to the relatively long life-expectancy of LGG patients, no post-mortem material was available to assess the micro-structural content for determining the correspondence with DWI-derived parameters. A direct comparison with histopathology would be required for determining the best parameter that reflects axonal fibre-bundle integrity in patients with LGG. Therefore, based on the low gradients found for DKI-FA and NODDI-FICVF in surgical cavity, the medium gradients in the peri-surgical cavity, and the high gradients in NAWM and WM, the decision could be based on the very low diffusivity observed for NODDI-FICVF in the surgical cavity as compared to DKI-FA. Another limitation could be our sample size. However, despite the small sample size, the findings of this study clearly indicated which methods correspond with biological expectations.

ADC is clinically widely used for the detection of tumours, as tumours have a high cellular density, and hence tumours reduce the Brownian motion of water molecules. While the usage of ADC has already been well established for tumour detection, DWI-derived micro-structural parameters are more representative of tissue integrity, such as axonal fibre-bundle integrity. As cognitive decline as a result of radiotherapy has been observed [5], there is a high need for a radiological biomarker quantification in early brain alterations. As cognition is amongst other a result of neuronal function, assessing axonal fibre-bundle integrity could function as such a biomarker. The evaluation of tissue micro-structural changes over time as derived from DWI in a longitudinal study enables a better understanding of the DWI parameters related to clinical outcomes, like cognition, tumour progression, and overall survival time. Also, exploring the role of the various DWI models in monitoring treatment-induced neurotoxicity (i.e., radiation-induced damage) would be of high interest. The agreement between both the various parameters related to ADC, like DTI-MD, DKI-MD, and WMTI-EAS-MD, and parameters related to axonal fibre-bundle integrity, suggest that the various DWI models can be used interchangeably (except for WMTI). However, whether the parameters assessing axonal fibre-bundle integrity might indeed be used as a biomarker for cognitive alterations remains to be investigated. Other techniques like MR spectroscopy (MRS) and [^11^C]UCB-J PET might also be interesting alternatives for assessing neuronal integrity. However, MRS has a long acquisition time (~20 min) and requires a priori selection of the brain region of investigation, and therefore cannot be used for whole-brain assessment. Current developments in MRS for whole-brain coverage are still in their infancy and are only available in highly specialized MRI centres. Meanwhile, [^11^C]UCB-J PET requires an acquisition time of 60 min and causes radiation exposure. Diffusion MRI, is in that sense, with a 6 min acquisition time and whole brain coverage, the most clinically interesting technique.

From a technical perspective, the DWI sequence required for ADC can be very fast (<2 min), as it represents the average diffusivity of water molecules in tissue, regardless of direction. Therefore, the number of diffusion directions for ADC calculation can be low (e.g., three directions). In contrast, the DWI sequence required for DTI, DKI, WMTI, NODDI, or FBA requires the acquisition of many diffusion directions (e.g., >32 directions). A higher number of directions captures a more complete diffusion signal, which might be different across different directions. The complex nature of the fibre architecture, with crossing fibres, kissing fibres, or branching fibres, requires an accurate measurement of the total diffusion signal. Hence, the acquisition of a high number of diffusion directions improves the parameter estimation with respect to the axonal fibre-bundle integrity. However, the acquisition of a high number of directions increases the scan duration, yet the sequence used in our study takes ~6 min, which is still a clinically acceptable acquisition time.

## 5. Conclusions

This study is the first to show a head-to-head comparison of DWI parameters derived from six different models within a single dataset. We found a high correspondence between the various DWI parameters, aside from WMTI-IAS-D_a_, with other axonal fibre-bundle integrity parameters. WMTI-IAS -D_a_ had a high signal within the surgical cavity and is thus not suitable. These findings suggests that all parameters relevant for tumour monitoring (ADC, MD, etc.) and all parameters for axonal fibre-bundle integrity (except for WMTI-IAS-D_a_) for assessing treatment-related changes could be used indistinguishably. As such, there would be a small preference for DTI over the other models due to the more computational and simpler analysis and, the on-scanner availability. The overall findings of this study are especially of importance due to the prolonged life expectancy of patients with LGG, and hence the increasing interest in measuring radiotherapy-induced damage in order to modify the existing therapies and help develop novel strategies to improve the cognitive status and quality of life of patients with LGG.

## Figures and Tables

**Table 1:** Overview of DWI parameters assessed in this study.

Model	Parameter	Abbreviation
Diffusion weighted imaging	Apparent diffusion coefficient	ADC
Diffusion tensor imaging	Axial diffusivity	DTI-AD
	Radial diffusivity	DTI-RD
	Mean diffusivity	DTI-MD
	Fractional anisotropy	DTI-FA
Diffusion kurtosis imaging	Axial diffusivity	DKI-AD
	Radial diffusivity	DKI-RD
	Mean diffusivity	DKI-MD
	Fractional anisotropy	DKI-FA
Fixel-based analysis	Fibre density	FBA-FD
White matter tract integrity	Extra-axonal space axial diffusivity	WMTI-EAS-AD
	Extra-axonal space radial diffusivity	WMTI-EAS-RD
	Extra-axonal space mean diffusivity	WMTI-EAS-MD
	Intra-axonal space, trace of axonal diffusion	WMTI-IAS-D_a_
	Axonal water fraction	WMTI-AWF
Neurite orientation dispersion and density imaging	Intra-cellular volume fraction (neurite density index)	NODDI-FICVF
	Isotropic volume fraction (free water fraction)	NODDI-FISO

**Table 2:** Subject characteristics.

	HC (N = 6)	LGG (N = 14)
Age in y (mean ± SD)	50.4 ± 4.9	46.5 ± 9.0
Gender (% male)	67%	57%
Tumour hemisphere (n per L/R)	n.a.	8/6
Radiotherapy (n per photon/proton)	n.a.	14/0
Radiotherapy dose in Gy (mean ± SD)	n.a.	52.6 ± 2.7
Chemotherapy (n per yes/no)	n.a.	12/2
End of radiotherapy to MRI scan interval in month (mean and range)	n.a.	39.1 (22–88)

Characteristics of the healthy controls (HC) and patients with lower-grade glioma (LGG) are shown. Abbreviations: Gy = Grey; L/R = left/right; SD = standard deviation; y = year.

**Table 3:** Comparison between DWI-derived parameters.

	H	Significance
Axonal fibre-bundle integrity	9609	>0.001
Axial diffusivity	2058	>0.001
Radial diffusivity	1406	>0.001
Mean diffusivity	9506	>0.001

The statistical results of all DWI-derived parameters are shown and are significantly different across gross tissue types like the surgical cavity, the peri-surgical cavity, normal-appearing white matter, and white matter. The H-value is the Kruskal–Wallis H-value.

## Data Availability

Due to privacy regulations, the clinical data collected in this study are not deposited in a public registry, but the data can be made available via a request to the corresponding author. Anonymized data can be made available after the approval of the participants and when a signed data transfer agreement is in place. The software programmes used in the study are publicly (FSL, MRtrix, ANTs, DKE, NODDI toolbox, PyDESIGNER) or commercially (PMOD, v4.1) available.

## Supplementary Materials

The following supporting information can be downloaded at https://www.mdpi.com/article/10.3390/jcm14020551/s1, Figure S1. Axonal fibre-bundle integrity parameters across different tissue types including WMTI-IAS-D_a_; Figure S2. Representative image of WMTI-IAS-D_a_; Figure S3. Correspondence between axonal fibre-bundle integrity parameters including WMTI-IAS-D_a_; Figure S4. Representative images of axial diffusivity related parameters. DTI-AD is the axial diffusivity as estimated using diffusion tensor imaging, DKI-AD is the axial diffusivity as estimated using diffusion kurtosis imaging, WMTI-EAS-AD is the extra-axonal space axial diffusivity as estimated using white matter tract integrity; Figure S5. Representative images of radial diffusivity related parameters. DTI-RD is the radial diffusivity as estimated using diffusion tensor imaging, DKI-RD is the radial diffusivity as estimated using diffusion kurtosis imaging, WMTI-EAS-RD is the extra-axonal space radial diffusivity as estimated using white matter tract integrity; Table S1. Assessing the potency of detecting tissue differences with DWI-derived parameters; Table S2. DWI parameters in surgical cavity, peri-surgical cavity, NAWM and WM; Table S3. Post-hoc analysis for the detection of tissue differences using DWI-derived axonal fibre-bundle integrity parameters; Table S4. Spearman correlations between axonal fibre-bundle integrity parameters; Table S5. ICC values between the axonal fibre-bundle integrity parameters using the two-way mixed model with absolute agreement; Table S6. Dunn’s post-hoc test for assessing differences between the DWI parameters; Table S7. Axial diffusivity related parameters; Table S8. Spearman correlations between axial diffusivity related parameters; Table S9. ICC values between the axial diffusivity related parameters using the two-way mixed model with absolute agreement; Table S10. Radial diffusivity related parameters; Table S11. Spearman correlations between radial diffusivity related parameters; Table S12. ICC values between the radial diffusivity related parameters using the two-way mixed model with absolute agreement; Table S13. Mean diffusivity related parameters; Table S14. Spearman correlations between mean diffusivity related parameters; Table S15. ICC values between the mean diffusivity related parameters using the two-way mixed model with absolute agreement.
