# Supplementary material for: Assessing the Validity of Diffusion Weighted Imaging Models: A Study in Patients with Post-Surgical Lower-Grade Glioma
Source: J Clin Med. 2025 Jan 16;14(2):551. doi: 10.3390/jcm14020551 (PMC11766432; doi:10.3390/jcm14020551)
Supplement: Supplementary file 1 [file jcm-14-00551-s001.zip › jcm-3385250-supplementary.pdf]

Journal of Clinical Medicine

Supplemental Materials

Table S1. Assessing the potency of detecting tissue differences with DWI-derived parameters.

Diffusion parameter	H	Significance
**Axonal-fibre bundle integrity**		
DTI-FA	41.5	**<0.001***
DKI-FA	42.0	**<0.001***
WMTI-AWF	41.6	**<0.001***
WMTI-IAS-D_a	40.9	**<0.001***
NODDI-FICVF-sticks	41.6	**<0.001***
NODDI-FICVF-cylinders	42.2	**<0.001***
FBA-FD	40.4	**<0.001***
**Axial diffusivity**		
DTI-AD	42.3	**<0.001***
DKI-AD	41.7	**<0.001***
WMTI-EAS-AD	32.7	**<0.001***
**Radial diffusivity**		
DTI-RD	42.7	**<0.001***
DKI-RD	42.9	**<0.001***
WMTI-EAS-RD	41.1	**<0.001***
**Mean diffusivity**		
ADC	42.9	**<0.001***
DTI-MD	42.7	**<0.001***
DKI-MD	42.7	**<0.001***
WMTI-EAS-MD	39.2	**<0.001***
NODDI-FISO-sticks	35.0	**<0.001***
NODDI-FISO-cylinders	34.9	**<0.001***

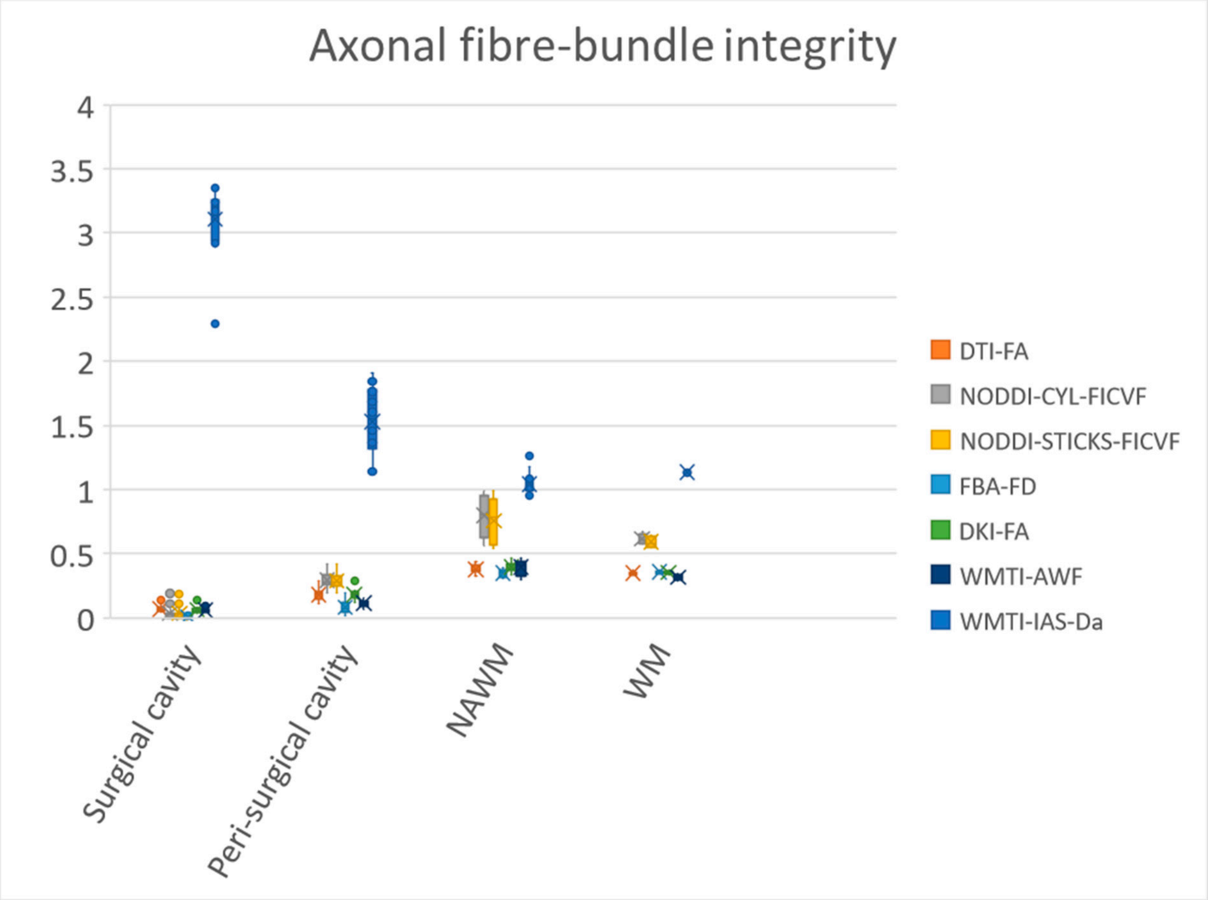

Figure S1. Axonal fibre-bundle integrity parameters across different tissue types including WMTI-IAS-D_a.

Table S2. DWI parameters in surgical cavity, peri-surgical cavity, NAWM and WM.

DWI method	Surgical cavity	Peri-surgical cavity	NAWM	WM
**Axonal-fibre bundle integrity**				
DTI-FA	0.07±0.03	0.18±0.05	0.38±0.03	0.35±0.01
DKI-FA	0.07±0.03	0.18±0.04	0.39±0.03	0.35±0.01
WMTI-AWF	0.06±0.01	0.11±0.03	0.39±0.05	0.32±0.02
WMTI-IAS-D_a	3.11±0.28	1.53±0.26	1.04±0.09	1.13±0.01
NODDI-FICVF-sticks	0.03±0.05	0.29±0.05	0.75±0.19	0.59±0.04
NODDI-FICVF-cylinders	0.04±0.06	0.29±0.05	0.80±0.17	0.62±0.04
FBA-FD	0.01±0.02	0.08±0.05	0.35±0.03	0.36±0.01
**Axial diffusivity**				
DTI-AD	3.02±0.25	1.58±0.28	1.03±0.07	1.10±0.02
DKI-AD	3.61±0.34	1.83±0.35	1.21±0.08	1.28±0.02
WMTI-EAS-AD	3.32±0.30	1.76±0.29	1.47±0.12	1.42±0.02
**Radial diffusivity**				
DTI-RD	2.74±0.29	1.22±0.22	0.56±0.05	0.63±0.02
DKI-RD	3.30±0.41	1.41±0.28	0.65±0.05	0.73±0.02
WMTI-EAS-RD	2.95±0.31	1.35±0.23	0.88±0.07	0.88±0.01

**Mean diffusivity**				
ADC	0.94±0.10	0.44±0.08	0.21±0.04	0.25±0.01
DTI-MD	2.83±0.27	1.34±0.24	0.72±0.05	0.79±0.02
DKI-MD	3.41±0.38	1.55±0.30	0.83±0.06	0.92±0.02
WMTI-EAS-MD	3.07±0.30	1.49±0.25	1.08±0.08	1.06±0.01
NODDI-FISO-sticks	0.93±0.11	0.30±0.19	0.10±0.10	0.08±0.04
NODDI-FISO-cylinders	0.93±0.11	0.30±0.19	0.10±0.10	0.08±0.04

Table S3. Post-hoc analysis for the detection of tissue differences using DWI-derived axonal fibre-bundle integrity parameters.

Diffusion parameter	Comparison	H	Significance
DTI-FA	Surgical cavity vs. peri-surgical cavity	13.3	0.067
	Surgical cavity vs. NAWM	32.6	**>0.001***
	Surgical cavity vs. WM	-26.4	**0.001***
	Peri-surgical cavity vs. NAWM	19.1	**0.002***
	Peri-surgical cavity vs. WM	-13.0	0.348
	NAWM vs. WM	-6.2	1.000
DKI-FA	Surgical cavity vs. peri-surgical cavity	13.4	0.067
	Surgical cavity vs. NAWM	33.0	**>0.001***
	Surgical cavity vs. WM	-25.4	**0.001***
	Peri-surgical cavity vs. NAWM	19.6	**0.001***
	Peri-surgical cavity vs. WM	-12.0	0.481
	NAWM vs. WM	7.6	1.000
WMTI-AWF	Surgical cavity vs. peri-surgical cavity	13.1	0.078
	Surgical cavity vs. NAWM	32.8	**>0.001***
	Surgical cavity vs. WM	-15.4	**0.001***
	Peri-surgical cavity vs. NAWM	19.6	**0.001***
	Peri-surgical cavity vs. WM	-12.3	0.436
	NAWM vs. WM	-7.4	1.000
WMTI-IAS-D_a	Surgical cavity vs. peri-surgical cavity	14.7	**0.033***
	Surgical cavity vs. NAWM	32.8	**>0.001***
	Surgical cavity vs. WM	-25.2	**0.001***
	Peri-surgical cavity vs. NAWM	18.1	**0.004***
	Peri-surgical cavity vs. WM	-10.5	0.756
	NAWM vs. WM	-7.6	1.000
NODDI-FICVF-sticks	Surgical cavity vs. peri-surgical cavity	14.0	**0.049***
	Surgical cavity vs. NAWM	32.2	**>0.001***
	Surgical cavity vs. WM	-28.2	**>0.001***
	Peri-surgical cavity vs. NAWM	18.2	**0.003***
	Peri-surgical cavity vs. WM	-14.2	0.229
	NAWM vs. WM	-4.0	1.000
NODDI-FICVF-cylinders	Surgical cavity vs. peri-surgical cavity	14.0	**0.049***

	Surgical cavity vs. NAWM	33.0	**>0.001***
	Surgical cavity vs. WM	-26.3	**0.001***
	Peri-surgical cavity vs. NAWM	19.0	**0.002***
	Peri-surgical cavity vs. WM	-12.3	0.426
	NAWM vs. WM	-6.7	1.000
FBA-FD	Surgical cavity vs. peri-surgical cavity	13.0	0.084
	Surgical cavity vs. NAWM	29.9	**>0.001***
	Surgical cavity vs. WM	-31.8	**>0.001***
	Peri-surgical cavity vs. NAWM	16.9	**0.008***
	Peri-surgical cavity vs. WM	-18.8	**0.035***
	NAWM vs. WM	-1.9	1.000

Table S4. Spearman correlations between axonal fibre-bundle integrity parameters.

	DTI-FA	DKI-FA	FBA-FD	WMTI-AWF	WMTI-IAS-D_a	NODDI -CYL-FICVF	NODDI -STICKS-FICVF
DTI-FA	x	0.995	0.912	0.901	0.229	0.875	0.873
DKI-FA	0.995	x	0.909	0.902	0.220	0.883	0.880
FBA-FD	0.912	0.909	x	0.769	0.296	0.831	0.826
WMTI-AWF	0.901	0.902	0.769	x	0.055	0.858	0.861
WMTI-IAS-D_a	0.229	0.220	0.296	0.055	x	0.067	0.049
NODDI -CYL-FICVF	0.875	0.883	0.831	0.858	0.067	x	0.995
NODDI -STICKS-FICVF	0.873	0.880	0.826	0.861	0.049	0.995	x

Table S5. ICC values between the axonal fibre-bundle integrity parameters using the two-way mixed model with absolute agreement.

	DTI-FA	DKI-FA	FBA-FD	WMTI-AWF	WMTI-IAS-D_a	NODDI-FICF-CYL	NODDI-FICF-STICKS
DTI-FA	x	0.995	0.873	0.853	0.009	0.164	0.164
DKI-FA	0.995	x	0.856	0.861	0.009	0.167	0.167
FBA-FD	0.873	0.856	x	0.626	0.012	0.147	0.144
WMTI-AWF	0.853	0.861	0.626	x	0.001	0.147	0.151
WMTI-IAS-D_a	0.009	0.009	0.012	0.001	x	-0.002	-0.004
NODDI-FICF-CYL	0.164	0.167	0.147	0.147	-0.002	x	0.984
NODDI-FICF-STICKS	0.164	0.167	0.144	0.151	-0.004	0.984	x

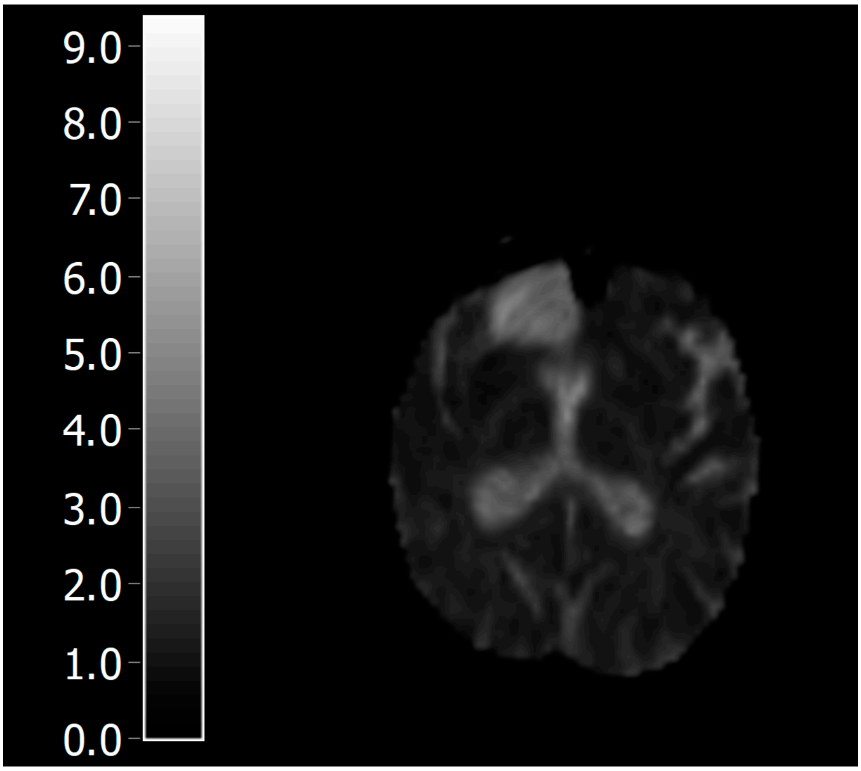

Figure S2. Representative image of WMTI-IAS-D_a.

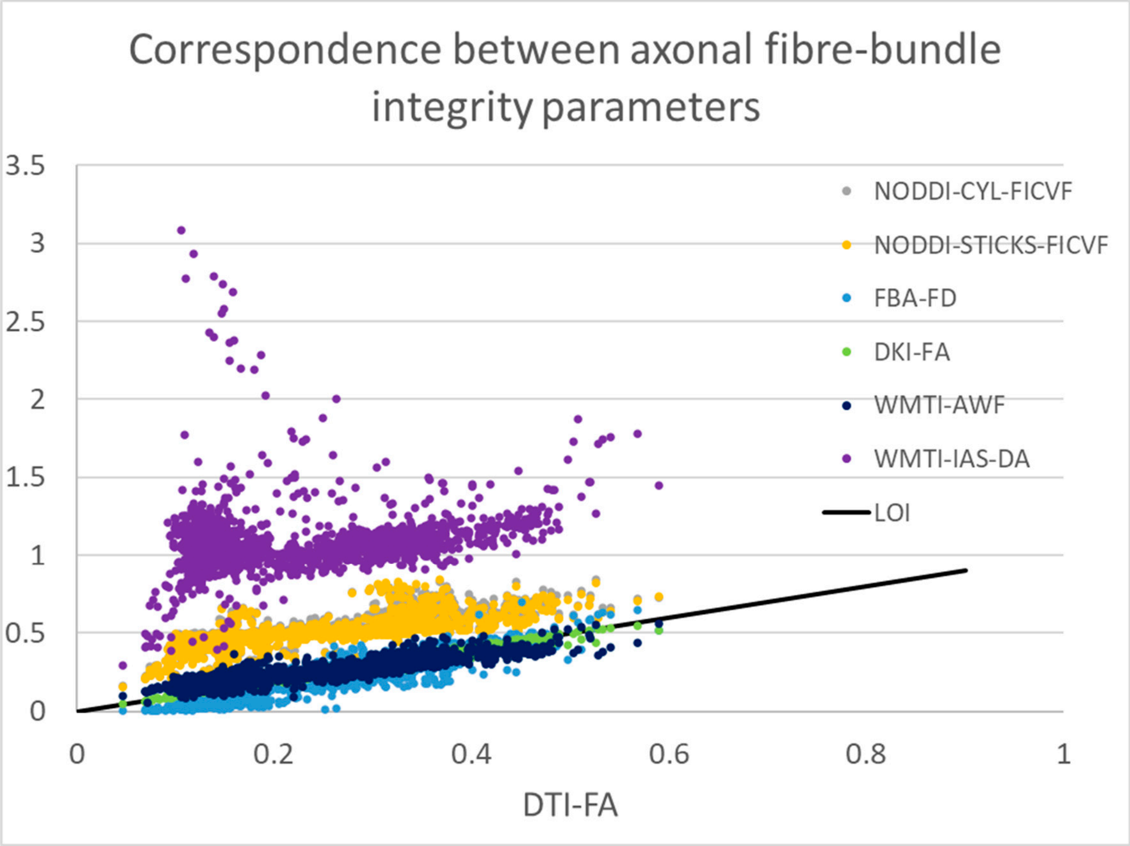

Figure S3. Correspondence between axonal fibre-bundle integrity parameters including WMTI-IAS-D_a.

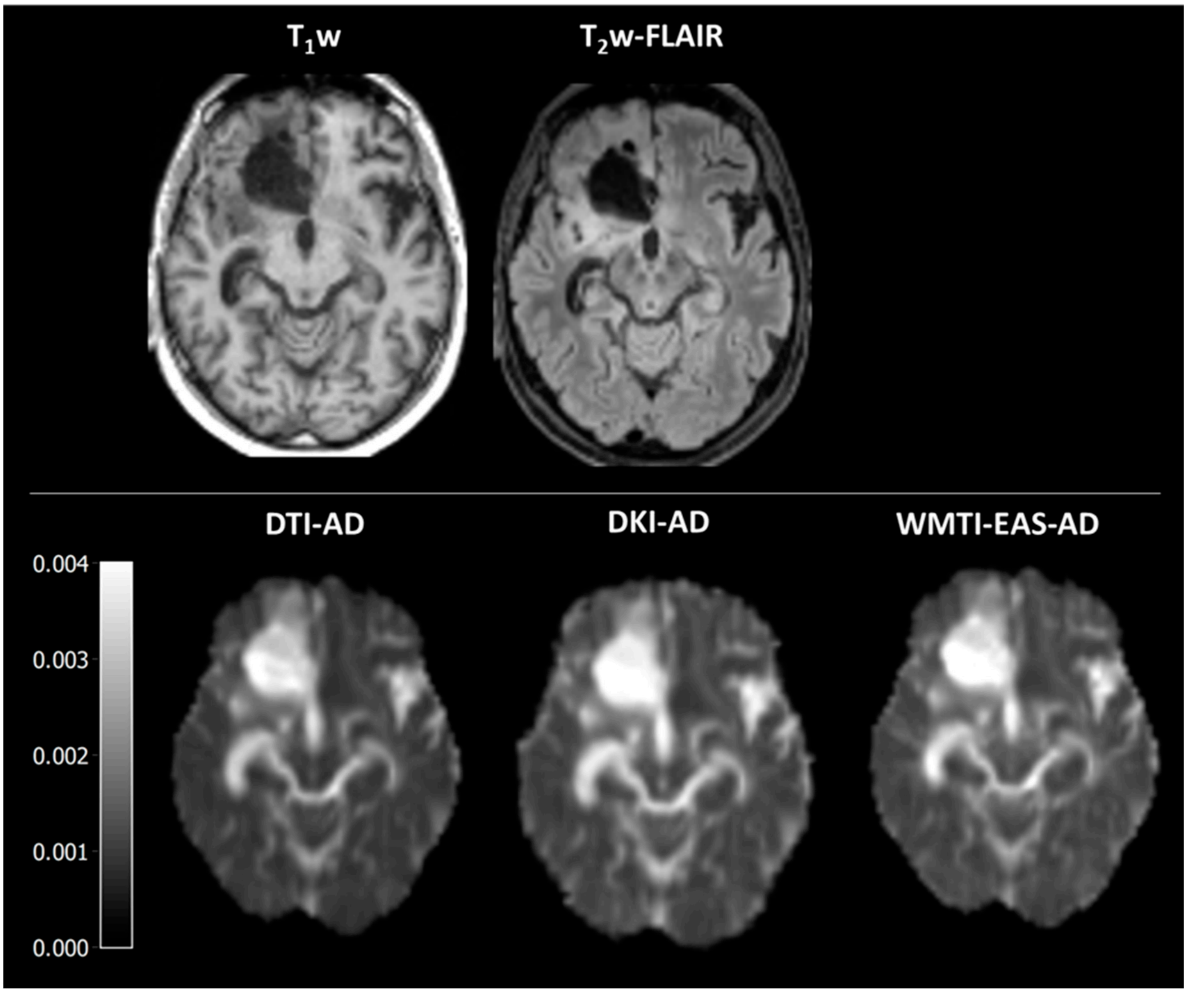

Figure S4. Representative images of axial diffusivity related parameters.*DTI-AD is the axial diffusivity as estimated using diffusion tensor imaging, DKI-AD is the axial diffusivity as estimated using diffusion kurtosis imaging, WMTI-EAS-AD is the extra-axonal space axial diffusivity as estimated using white matter tract integrity.*

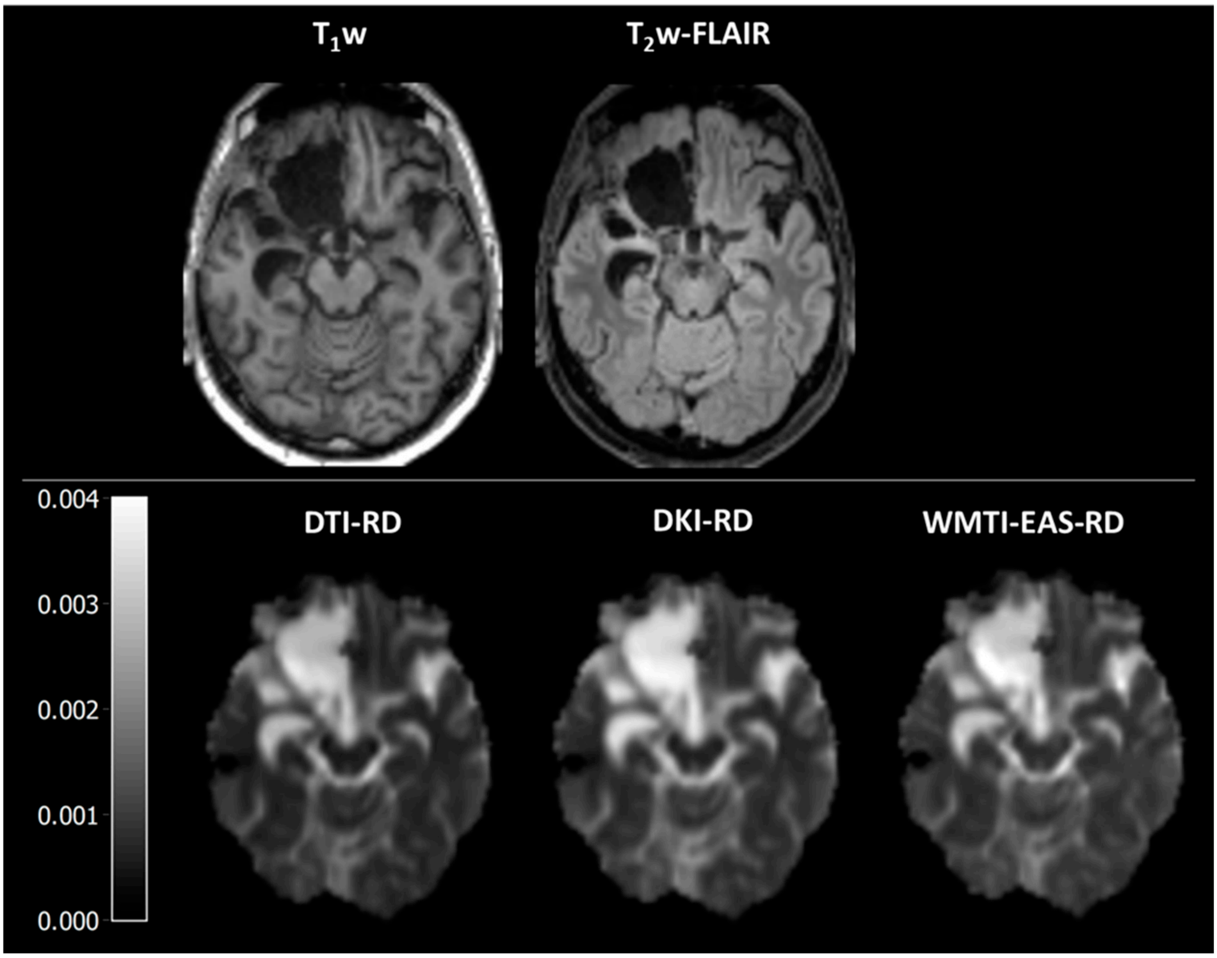

Figure S5. Representative images of radial diffusivity related parameters. *DTI-RD is the radial diffusivity as estimated using diffusion tensor imaging, DKI-RD is the radial diffusivity as estimated using diffusion kurtosis imaging, WMTI-EAS-RD is the extra-axonal space radial diffusivity as estimated using white matter tract integrity.*

Table S6. Dunn's post-hoc test for assessing differences between the DWI parameters.

Comparison	Test statistic (H)	Adjusted Significance
**Axonal fibre-bundle integrity**		
FBA-FD vs. DTI-FA	721	**>0.001***
FBA-FD vs. DKI-FA	847	**>0.001***
FBA-FD vs. WMTI-AWF	-1204	**>0.001***
FBA-FD vs. NODDI-STICKS-FICVF	-5825	**>0.001***
FBA-FD vs. NODDI-CYL-FICVF	-5988	**>0.001***
FBA-FD vs. WMTI-IAS-D_a	-8808	**>0.001***
DTI-FA vs. DKI-FA	126	1.000
DTI-FA vs. WMTI-AWF	-483	**0.002***
DTI-FA vs. NODDI-STICKS-FICVF	-5104	**>0.001***
DTI-FA vs. NODDI-CYL-FICVF	-5267	**>0.001***
DTI-FA vs. WMTI-IAS-D_a	-8088	**>0.001***
DKI-FA vs. WMTI-AWF	-357	0.074
DKI-FA vs. NODDI-STICKS-FICVF	-4979	**>0.001***

DKI-FA vs. NODDI-CYL-FICVF	-5141	**>0.001***
DKI-FA vs. WMTI-IAS-D_a	-7962	**>0.001***
WMTI-AWF vs. NODDI-STICKS-FICVF	4622	**>0.001***
WMTI-AWF vs. NODDI-CYL-FICVF	4784	**>0.001***
WMTI-AWF vs. WMTI-IAS-D_a	-7605	**>0.001***
NODDI-STICKS-FICVF vs. NODDI-CYL-FICVF	162	1.000
NODDI-STICKS-FICVF vs. WMTI-IAS-D_a	-2983	**>0.001***
NODDI-CYL-FICVF vs. WMTI-IAS-D_a	-2821	**>0.001***
**Axial diffusivity**		
DTI-AD vs. DKI-AD	1639	**>0.001***
DTI-AD vs. WMTI-EAS-AD	-2312	**>0.001***
DKI-AD vs. WMTI-EAS-AD	-673	**>0.001***
**Radial diffusivity**		
DTI-RD vs. DKI-RD	1073	**>0.001***
DTI-RD vs. WMTI-EAS-RD	-1963	**>0.001***
DKI-RD vs. WMTI-EAS-RD	-890	**>0.001***
**Mean diffusivity**		
NODDI-CYL-FISO vs. NODDI-STICKS-FISO	-14	1.000
NODDI-CYL-FISO vs. ADC	2577	**>0.001***
NODDI-CYL-FISO vs. DTI-MD	5106	**>0.001***
NODDI-CYL-FISO vs. DKI-MD	6496	**>0.001***
NODDI-CYL-FISO vs. WMTI-EAS-MD	-7438	**>0.001***
NODDI-STICKS-FISO vs. ADC	2563	**>0.001***
NODDI-STICKS-FISO vs. DTI-MD	5092	**>0.001***
NODDI-STICKS-FISO vs. DKI-MD	6482	**>0.001***
NODDI-STICKS-FISO vs. WMTI-EAS-MD	-7424	**>0.001***
ADC vs. DTI-MD	2528	**>0.001***
ADC vs. DKI-MD	3918	**>0.001***
ADC vs. WMTI-EAS-MD	-4861	**>0.001***
DTI-MD vs. DKI-MD	1390	**>0.001***
DTI-MD vs. WMTI-EAS-MD	-2332	**>0.001***
DKI-MD vs. WMTI-EAS-MD	-942	**>0.001***

Table S7. Axial diffusivity related parameters.

Diffusion parameter	Comparison	H	Significance
DTI-AD	Surgical cavity vs. peri-surgical cavity	14.1	**0.047***
	Surgical cavity vs. NAWM	33.2	**>0.001***
	Surgical cavity vs. WM	-25.8	**0.001***
	Peri-surgical cavity vs. NAWM	19.1	**0.002***
	Peri-surgical cavity vs. WM	-11.7	0.524
	NAWM vs. WM	-7.4	1.000
DKI-AD	Surgical cavity vs. peri-surgical cavity	14.2	**0.043***
	Surgical cavity vs. NAWM	32.9	**>0.001***
	Surgical cavity vs. WM	-26.2	**>0.001***
	Peri-surgical cavity vs. NAWM	18.6	**0.003***
	Peri-surgical cavity vs. WM	-12.0	0.481
	NAWM vs. WM	-6.7	1.000
WMTI-EAS-AD	Surgical cavity vs. peri-surgical cavity	18.6	**0.003***
	Surgical cavity vs. NAWM	27.4	**>0.001***
	Surgical cavity vs. WM	-28.7	**>0.001***
	Peri-surgical cavity vs. NAWM	8.7	0.598
	Peri-surgical cavity vs. WM	-10.0	0.854
	NAWM vs. WM	-1.3	1.000

Table S8. Spearman correlations between axial diffusivity related parameters.

	DTI-AD	DKI-AD	WMTI-EAS-AD
DTI-AD	x	0.965	0.837
DKI-AD	0.965	x	0.809
WMTI-EAS-AD	0.837	0.809	x

Table S9. ICC values between the axial diffusivity related parameters using the two-way mixed model with absolute agreement.

	DTI-AD	DKI-AD	WMTI-EAS-AD
DTI-AD	x	0.744	0.558
DKI-AD	0.744	x	0.876
WMTI-EAS-AD	0.558	0.876	x

Table S10. Radial diffusivity related parameters.

Diffusion parameter	Comparison	H	Significance
DTI-RD	Surgical cavity vs. peri-surgical cavity	14.0	**0.049***
	Surgical cavity vs. NAWM	33.4	**>0.001***
	Surgical cavity vs. WM	-25.3	**0.001***
	Peri-surgical cavity vs. NAWM	19.4	**0.001***
	Peri-surgical cavity vs. WM	-11.3	0.583
	NAWM vs. WM	-8.1	1.000

DKI-RD	Surgical cavity vs. peri-surgical cavity	14.0	**0.049***
	Surgical cavity vs. NAWM	33.6	**>0.001***
	Surgical cavity vs. WM	-24.8	**0.002***
	Peri-surgical cavity vs. NAWM	19.6	**0.001***
	Peri-surgical cavity vs. WM	-10.8	0.677
	NAWM vs. WM	-8.8	1.000
WMTI-EAS-RD	Surgical cavity vs. peri-surgical cavity	14.1	**0.047***
	Surgical cavity vs. NAWM	30.6	**>0.001***
	Surgical cavity vs. WM	-31.8	**>0.001***
	Peri-surgical cavity vs. NAWM	16.5	**0.011***
	Peri-surgical cavity vs. WM	-17.8	0.056
	NAWM vs. WM	-1.3	1.000

Table S11. Spearman correlations between radial diffusivity related parameters.

	DTI-RD	DKI-RD	WMTI-EAS-RD
DTI-RD	x	0.979	0.851
DKI-RD	0.979	x	0.869
WMTI-EAS-RD	0.851	0.869	x

Table S12. ICC values between the radial diffusivity related parameters using the two-way mixed model with absolute agreement.

	DTI-RD	DKI-RD	WMTI-EAS-RD
DTI-RD	x	0.834	0.606
DKI-RD	0.834	x	0.861
WMTI-EAS-RD	0.606	0.861	x

Table S13. Mean diffusivity related parameters.

Diffusion parameter	Comparison	H	Significance
ADC	Surgical cavity vs. peri-surgical cavity	14.0	**0.049***
	Surgical cavity vs. NAWM	33.6	**>0.001***
	Surgical cavity vs. WM	-25.0	**0.002***
	Peri-surgical cavity vs. NAWM	19.6	**0.001***
	Peri-surgical cavity vs. WM	-11.0	0.644
	NAWM vs. WM	-8.6	1.000
DTI-MD	Surgical cavity vs. peri-surgical cavity	14.0	**0.049***
	Surgical cavity vs. NAWM	33.4	**>0.001***
	Surgical cavity vs. WM	-25.3	**0.001***
	Peri-surgical cavity vs. NAWM	19.4	**0.001***
	Peri-surgical cavity vs. WM	-14.0	0.583
	NAWM vs. WM	-8.1	1.000
DKI-MD	Surgical cavity vs. peri-surgical cavity	14.0	**0.049***
	Surgical cavity vs. NAWM	33.4	**>0.001***
	Surgical cavity vs. WM	-25.3	**0.001***

	Peri-surgical cavity vs. NAWM<br>Peri-surgical cavity vs. WM<br>NAWM vs. WM	19.4<br>-11.3<br>-8.1	**0.001***<br>0.583<br>1.000
WMTI-EAS-MD	Surgical cavity vs. peri-surgical cavity<br>Surgical cavity vs. NAWM<br>Surgical cavity vs. WM<br>Peri-surgical cavity vs. NAWM<br>Peri-surgical cavity vs. WM<br>NAWM vs. WM	14.9<br>29.9<br>-31.5<br>14.9<br>-16.6<br>-1.6	**0.029***<br>**>0.001***<br>**>0.001***<br>**0.029***<br>0.092<br>1.000
NODDI-FISO-sticks	Surgical cavity vs. peri-surgical cavity<br>Surgical cavity vs. NAWM<br>Surgical cavity vs. WM<br>Peri-surgical cavity vs. NAWM<br>Peri-surgical cavity vs. WM<br>NAWM vs. WM	16.6<br>28.9<br>-28.9<br>12.3<br>-12.3<br>0.0	**0.010***<br>**>0.001***<br>**>0.001***<br>0.121<br>0.432<br>1.000
NODDI-FISO-cylinders	Surgical cavity vs. peri-surgical cavity<br>Surgical cavity vs. NAWM<br>Surgical cavity vs. WM<br>Peri-surgical cavity vs. NAWM<br>Peri-surgical cavity vs. WM<br>NAWM vs. WM	16.6<br>28.6<br>-29.2<br>12.0<br>-12.5<br>-0.5	**0.010***<br>**>0.001***<br>**>0.001***<br>0.140<br>0.397<br>1.000

Table S14. Spearman correlations between mean diffusivity related parameters.

	DWI-ADC	DTI-MD	DKI-MD	WMTI-EAS-MD	NODDI-CYL-FISO	NODDI-STICKS-FISO
DWI-ADC	x	0.837	0.815	0.625	0.612	0.621
DTI-MD	0.837	x	0.963	0.742	0.680	0.689
DKI-MD	0.815	0.963	x	0.763	0.726	0.730
WMTI-EAS-MD	0.625	0.742	0.763	x	0.706	0.693
NODDI-CYL-FISO	0.612	0.680	0.726	0.706	x	0.989
NODDI-STICKS-FISO	0.621	0.689	0.730	0.693	0.989	x

Table S15. ICC values between the mean diffusivity related parameters using the two-way mixed model with absolute agreement.

	DWI-ADC	DTI-MD	DKI-MD	WMTI-EAS-MD	NODDI-CYL-FISO	NODDI-STICKS-FISO
DWI-ADC	x	0.063	0.049	0.032	0.216	0.218

DTI-MD	0.063	x	0.785	0.554	0.049	0.049
DKI-MD	0.049	0.785	x	0.851	0.043	0.043
WMTI-EAS-MD	0.032	0.554	0.851	x	0.031	0.031
NODDI-CYL-FISO	0.216	0.049	0.043	0.031	x	0.995
NODDI-STICKS-FISO	0.218	0.049	0.043	0.031	0.995	x
